# Challenges and solutions in hand pollination for hybrid pepper (*Capsicum annuum* L.) seed production: a review

**DOI:** 10.1007/s00425-026-05012-z

**Published:** 2026-04-24

**Authors:** Ingrid M. Gyalai, Orsolya Kedves, Alfonz Kedves, Zoltán Kónya, Flórián Kovács

**Affiliations:** 1 Faculty of Agriculture, Institute of Plant Sciences and Environmental Protection, University of Szeged,, Andrássy Út 15, 6800 Hódmezővásárhely, Hungary; 2 Department of Biotechnology and Microbiology, Faculty of Science and Informatics, University of Szeged, Szeged, Hungary; 3 Department of Applied and Environmental Chemistry, University of Szeged, Szeged, Hungary; 4 Institute of Animal Science and Wildlife Management, Faculty of Agriculture, University of Szeged, Hódmezővásárhely, Hungary

**Keywords:** Pollen, Heat sensitivity, Hormonal regulation, Hand pollination, Genetic factors

## Abstract

**Main conclusion:**

**Successful hybrid seed production in pepper depends strongly on the stress sensitivity of reproductive processes, particularly pollen function and flower retention. Integrating physiological understanding of pollen biology, hormonal regulation of abscission, and targeted environmental and genetic interventions is essential to stabilize fruit set and improve hybrid seed yield under increasing climatic variability.**

**Abstract:**

Hybrid pepper (*Capsicum annuum* L.) F_1_ seed production relies largely on controlled (manual) emasculation and pollination, while seed set at commercial scale is often unstable due to the pronounced environmental sensitivity of the reproductive phase. The present review discusses, within an integrated framework, the main constraints of manual pollination and hybrid seed yield as well as the possible solutions, with particular emphasis on (i) abiotic stressors (temperature, light intensity and light spectrum, water and nutrient supply, relative humidity), (ii) pollen biological and progamic processes, (iii) the hormonal regulation of flower and young fruit abscission, and (iv) genetic/male-sterility systems supporting hybrid purity. Based on the literature, pollen is the most stress-sensitive “weak link”: consistently high temperatures (above 32 °C) and unfavorable light regimes impair pollen development, viability, and pollen tube growth, while shifts in the auxin-ethylene balance in the abscission zone increase flower and fruit drop. Reduced assimilate availability (source-sink competition) and the hormonal dominance of developing fruits further intensify abortion, in protected cultivation potentially leading to cyclic fruit-set patterns. Although CMS/CGMS (cytoplasmic and cytoplasmic-genic male sterility) and GMS (genic male sterility) systems can reduce labor costs and improve genetic purity, their application is not suitable in all breeding and hybrid seed production scenarios; therefore, controlled pollination performed with manual emasculation remains of key importance. The review proposes a physiology-based, decision-support approach that integrates microclimate optimization (thermal and spectral management), pollen-based rapid phenotyping, and marker-based male-sterility identification to improve successful fertilization, seed formation, and hybrid seed quality. In this review, the most critical research gap is the lack of an empirically validated relationship between in vitro pollen stress assays and in vivo fertilization and seed-set success, as this could establish the predictive foundations of stress-tolerant, scalable hybrid seed production.

**Graphical abstract:**

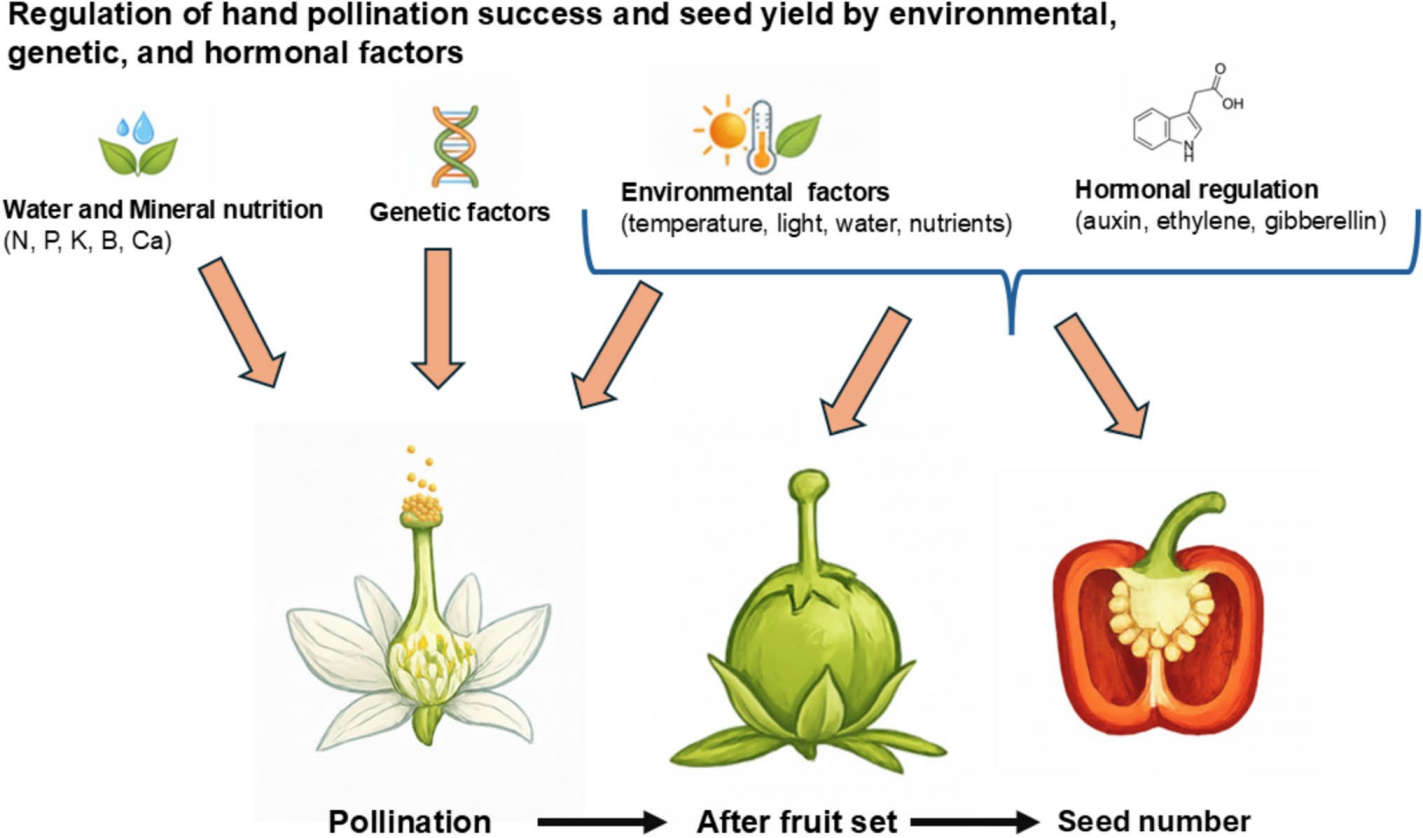

## Introduction

Peppers (*Capsicum annuum* L.) are widely appreciated and consumed worldwide, with global production reaching approximately 5.8 million tons in 2023 (FAO 2025). During their extensive cultivation, plants face numerous abiotic challenges (Hosseinifard et al. 2022). The pepper seed market represents a substantial economic sector, clearly reflecting the prominent industrial importance of hybrid seed production within the cultivation chain (Tripodi et al. 2021; Naves et al. 2022). According to estimates of the Intergovernmental Panel on Climate Change (IPCC), the global average temperature is expected to increase by 3.7–4.8 °C by 2100 (Mumtaz et al. 2023), making the generative development, pollination, and fruit set of pepper particularly vulnerable. In addition to extreme abiotic factors, genetic diversity is a key requirement in every plant breeding program (Sood et al. 2023). F_1_hybrid cultivars are widely used in pepper production, as this species exhibits pronounced heterosis (Guo et al. 2014). It is estimated that hybrids account for 80% of all pepper seeds used. F_1_hybrids exhibit more uniform growth and generally achieve higher yields by 15–50%—depending on crop type—than parental lines (Fu et al. 2014; Meena et al. 2020). The production of F_1_hybrid bell pepper seeds is one of the most profitable sectors worldwide; therefore, there is a strong demand for efficient and cost-effective approaches in commercial hybrid seed production (Cheng et al. 2018).

Currently, hybrid seed production largely relies on labor-intensive manual pollination and emasculation. In contrast, seed production based on male sterility can reduce costs, as it requires less labor and time for the preparation of female flowers. In addition, the application of male sterility can increase the genetic purity of F_1_seeds by preventing self-pollination (Wang et al. 2006). Although cytoplasmic male sterility plays an important role in pepper hybrid production, its underlying molecular mechanisms remain incompletely understood (Zhang et al. 2015). Several DNA markers specific to cytoplasmic male sterility (Kim and Kim 2005), as well as SCAR (Sequence Characterized Amplified Region) markers derived from mitochondrial sequences, have been developed (Kim and Kim 2006); however, their practical application may be limited, as PCR amplification may occasionally occur at a low level in maintainer lines as well (Min 2009). Nevertheless, male sterility cannot be applied universally, particularly in breeding programs and in the production of specific hybrids; therefore, controlled pollination performed through manual emasculation continues to play an important role in pepper hybrid seed production (Shifriss 1997; Dhaliwal & Jindal 2014a, b).

The aim of this review is to discuss, within an integrated framework, the major abiotic stressors (heat, light, and water/nutrient supply) determining pepper fruit set and hybrid seed production, pollen biology and hormonal regulation, and the relevant findings on genetic and male-sterility systems, with particular emphasis on flower and fruit abscission, seed formation, and parthenocarpy.

## Heat stress and reduced reproductive performance

Heat stress has numerous detrimental effects on plants, including protein denaturation, suppression of protein synthesis, protein degradation, and changes in the fluidity and integrity of lipid membranes (Howarth 2005; Zhang et al. 2022). The impact of heat stress varies among crops, species, and cultivars (Sita et al. 2017a, b), partly due to differences in ecological adaptation and species-specific temperature optima (Yamamoto et al. 2011). The effect of high temperature depends on the intensity and duration of the stress, fluctuations above the optimal temperature, and the developmental stage of the plant (Alghabari et al. 2015).

In pepper, heat stress can cause substantial yield losses during the vegetative and, most critically, the generative (reproductive) stages (Kafizadeh et al. 2008). Temperatures approximately 32 °C can severely impair pollination and fertilization, leading to reduced fruit set or complete fruit-set failure (Pérez-Jiménez et al. 2019). Gisbert-Mullor et al. (2021) determined that the pollen viability of flower buds smaller than 2.5 mm greatly decreased when they were exposed to 33 °C for 120 h. Plant responses to high temperature include short-term stress avoidance or acclimation mechanisms, such as leaf repositioning, evaporative cooling through transpiration, and modification of membrane lipid composition (Wahid et al. 2007). Exposure to moderately elevated temperatures may induce acclimation, conferring acquired thermotolerance against subsequent severe heat stress (Wahid et al. 2007; Senthil-Kumar et al. 2007).

Yamazaki and Hosokawa (2019) reported that the inbred C. chinense lines ‘Sy-2’ and ‘No. 3686’ showed a strong reduction in fruit set under high-temperature conditions, whereas their F1 hybrid maintained a high fruit set. Reciprocal artificial pollination experiments indicated that both female and male factors contribute to the enhanced fruit-setting capacity of the F1, which was further supported by a reduced heat-induced decline in pollen germination in the hybrid.

Generative organs are generally more heat-sensitive to heat stress than vegetative tissues; therefore, temperature stress occurring during the reproductive phase can have a disproportionately large impact on fruit set and yield (Hedhly and Hormaza, 2009; Jagadish et al. 2010a, b; Sita et al. 2017a, b). The reproductive response to heat stress involves several stages with differing sensitivities, including gametogenesis, the progamic phase from pollination to fertilization, and early embryo development. High temperatures often promote flower and young fruit abortion (abscission), which represents a direct physiological cause of yield loss (Warner & Erwin 2005; Koti 2005). In pepper, yield reduction induced by heat stress is typically not primarily associated with impaired vegetative growth, but rather with disturbances in reproductive processes—particularly pollen development, pollination, and early fruit retention (Peet et al. 1998; Pressman et al. 2002a, b; Wubs et al. 2009a, b).

### Pollen as a critical “weak link”

Pollen is one of the most heat-sensitive plant tissues (Hedhly et al. 2009; Giorno et al. 2013). Consistently high temperatures impair pollen development and metabolism, reduce viability and germination capacity (Aloni et al. 2001; Pressman et al. 2002a, b; Firon et al. 2006; Wubs et al. 2009a, b; Müller & Rieu 2016; Kotak et al. 2007), and thereby directly decrease fruit set. Fruit set itself can serve as a screening trait for heat tolerance; however, it does not allow separate assessment of individual floral and pollen parameters and is highly influenced by environmental variability. The heat sensitivity of pollen is manifested not only in reduced germination rates but also in a reduction in pollen tube growth rate and the occurrence of structural and morphological abnormalities, which further decrease the probability of successful fertilization (Hedhly et al. 2004; Sato et al. 2002a, b; Müller & Rieu 2016).

In vitro pollen heat-stress assays have proven to be effective tools for evaluating thermotolerance. For example, Heidmann et al. (2016) demonstrated that the tomato pollen germination was examined between 37 and 40 °C, and it was shown that heat treatment at 38 °C negatively affected pollen germination in tobacco and tomato, which is consistent with previous publications. However, in pepper, only limited empirical evidence is available that directly links in vitro heat-stress responses to in vivo pollination, fertilization, and seed-set success. Moreover, the relationship between in vitro pollen traits and in vivo fruit set is often non-linear, as the physiological status of female tissues, processes occurring during the progamic phase, and interactions with fluctuating environmental conditions can substantially modify fertilization success (Hedhly et al. 2004; Peet et al. 1998; Wubs et al. 2009a, b).

### Breeding for heat tolerance: complex genetic background and the role of wild relatives

Managing heat stress in cultivation is challenging; therefore, genetic improvement for heat tolerance is regarded as one of the most effective long-term strategies, although it requires the simultaneous evaluation of multiple physiological and reproductive traits (Lin et al. 2021). Wild relative species represent important genetic resources for improving heat tolerance (Driedonks et al. 2018a, b). Heat tolerance is a complex, polygenic trait resulting from the interaction of multiple genes (Jagadish et al. 2010a, b; Grover et al. 2013; Opeña et al. 1992; Lucas et al. 2013; Dickson 1992), and the expressed phenotype is often strongly dependent on the applied temperature regime (Ye et al. 2012).

Breeding for heat tolerance is particularly challenging during the reproductive phase, as generative traits—such as pollen viability, fruit set, and seed formation—are highly sensitive to environmental variation and often exhibit low to moderate heritability (Jagadish et al. 2010a, b; Müller & Rieu 2016; Sita et al. 2017a, b).

The substantial genetic diversity of wild *Capsicum* species and their adaptation to extreme environments are promising; however, introgression breeding in pepper has progressed more slowly than in other *Solanaceae* species, partly due to crossing barriers, reduced fertility in interspecific hybrids, and linkage drag (Aguilar et al. 2009; Carrizo García et al. 2016; Cheng et al. 2016; Oyama et al. 2006; Votava et al. 2002; Khoury et al. 2020; Parry et al. 2021).

Recent QTL-driven studies in Capsicum challenge the traditionally pollen-focused framework of reproductive heat tolerance. Yamazaki et al. (2025) identified a major-effect locus, qFS6 on chromosome 6, that was robustly associated with enhanced fruit set under elevated temperature conditions in greenhouse experiments yet showed no detectable linkage to pollen viability or dispersal traits. This decoupling indicates that, in certain genetic contexts, improved reproductive success under heat stress may arise through mechanisms other than male gametophytic function, highlighting the potential importance of processes such as ovary persistence and the regulation of abscission responses.

Nevertheless, several studies have demonstrated that alleles derived from wild relatives can effectively enhance heat tolerance, particularly when selection focuses on reproductive performance-related phenotypes, such as pollen viability, fruit set, and seed retention under elevated temperatures (Driedonks et al. 2018a, b; Jagadish et al. 2016; Tripodi et al. 2021). Key genes and genomic regions associated with reproductive heat tolerance in pepper and related *Capsicum* species are summarized in Table 1.

**Table 1:** Genes and genomic regions associated with reproductive heat tolerance in pepper and related *Capsicum* species

Functional category	Gene/genomic region	Species/source	Reproductive trait affected	Physiological role under heat stress	References
Heat shock response	HSP70	*Capsicum annuum*	Pollen viability	Stabilization of proteins and maintenance of cellular metabolism during pollen development under elevated temperature	Pressman et al. 2002a, b
Heat shock response	Small heat shock proteins (sHSPs)	*Capsicum annuum*	Pollen development	Prevention of protein aggregation and protection of pollen cellular integrity during heat stress	Firon et al. 2006
Transcriptional regulation	HSFA2	*Solanum lycopersicum* (Solanaceae model)	Fruit set	Activation of heat-responsive transcriptional networks in reproductive tissues	Jagadish et al. 2010a, b
Oxidative stress regulation	APX (ascorbate peroxidase)	*Capsicum annuum*	Pollen germination	ROS detoxification, limiting oxidative damage in pollen under heat stress	Sita et al. 2017a, b
Carbohydrate metabolism	Cell wall invertase (INV)	*Capsicum annuum*	Pollen tube growth	Maintenance of carbohydrate supply required for pollen tube elongation and fertilization under heat stress	Firon et al. 2006
Hormonal homeostasis	GA2ox	*Capsicum annuum*	Fruit set, seed formation	Regulation of gibberellin levels influencing reproductive organ retention and early fruit development under stress	Tripodi et al. 2021
Reproductive development (stress-sensitive node)	CaOFP20 (Ovate Family Protein)	*Capsicum annuum*	Male fertility, pollen formation	Regulation of floral organ development and male fertility; altered function can exacerbate reproductive failure under heat stress	Cheng et al. 2018
Wild introgression loci	Heat tolerance–related QTLs	*C. chacoense, C. baccatum*	Fruit set	Enhancement of reproductive stability at elevated temperature through introgressed alleles from wild relatives	Driedonks et al. 2018a, b; Tripodi et al. 2021

## Light intensity and light spectrum: fruit set, assimilate supply, and hormonal regulation

The spectral composition of light plays a key role in fruit set. A low red:far-red (R:FR) ratio or a very high blue:red (B:R) ratio reduces the phytochrome photostationary state (PSS), which in turn promotes flower and fruit drop in sweet pepper (Chen et al. 2022; Chen et al. 2024a, b; Legris et al. 2019; Kong et al. 2018, 2019). Flower and fruit drop represent hormonally regulated abscission processes, in which ethylene and abscisic acid act as promoters, whereas auxin and gibberellin have inhibitory roles (Crane 1969; Taylor and Whitelaw 2001; Sawicki et al. 2015). A high B:R ratio may also influence fruit set through light-dependent modulation of hormonal balance, partly mediated by blue-light receptors (Chen et al. 2024a, b).

Light intensity is one of the primary determinants of generative organ retention (Ascough et al. 2005). Reduced light intensity and shading increase flower abscission and reduce fruit formation in many crop species. According to Ferree et al. (2001), five days of 80% shading during flowering reduced grape fruit set from 47.3% to 29.2%. Zhu et al. (2011) found that, after 19 days of shading, about 98% of the young fruits abscised, whereas under normal (control) conditions less than 10% dropped. According to Aloni et al. (1996), for example, in some cultivars flower abscission increased to 86–100% when the maximum light intensity was reduced from 920 µmol m⁻^2^ s⁻^1^ to 200 µmol m⁻^2^ s⁻^1^. The effects of photoperiod and light spectrum are often closely coupled with total light quantity, making their individual contributions difficult to separate (Demers et al. 1998).

Fruit sets are also strongly dependent on carbohydrate availability. Low sugar and starch contents promote flower abscission (Aloni et al. 1996), whereas supplemental sugar supply can reduce abscission, partly through the activity of key enzymes involved in sucrose metabolism, such as sucrose synthases and invertases (Aloni et al. 1997; Nielsen et al. 1991; Ruan et al. 2012). A very high proportion of blue light can reduce leaf starch accumulation and overall dry matter production, thereby limiting fruit set through restricted photoassimilate supply (Warrington & Mitchell 1976; Wang et al. 2016; He et al. 2017; Larsen et al. 2022).

Mechanistic processes underlying fruit abortion under far-red (FR) light include reduced sucrose accumulation, altered invertase activity, and interactions between carbohydrate status and hormonal signaling pathways (Li et al. 2022; Chen et al. 2024a, b).

## Pollen, flower biology, and fruit set: fundamentals and stress sensitivity

### Flower biology, pollen production, and seed formation

Pollen is a central component of plant reproduction, and its long-term, viability and preservation are crucial in breeding and genetic resource maintenance programs. Pepper pollen is relatively short-lived, making cryopreservation relevant in some programs (Ren et al. 2019; Jia et al. 2022). Pepper is often partially cross-pollinated (7–65% outcrossing) (Herath et al. 2020), and the availability of male-sterility systems supports the widespread use of hybrids cultivars.

Successful fruit set depends on the synchronization of anthesis, pollen viability and germination capacity, and stigma receptivity (Peña-Yam et al. 2019; Dhall et al. 2011). Pollen production often correlates with another characteristic and may be closely associated with seed formation; high pollen viability increases the likelihood of successful pollination (Agnihotri and Singh 1975; Dafni 1992; Minnaar et al. 2019). Experiments have demonstrated that both open and manual pollination can increase fruit set, fruit size, and seed number compared to controls (Bergonia and Cervancia 1992; Deprá et al. 2014; Krell 2018).

Seed set can serve as a direct indicator for pollen fertilization success as incomplete fertilization can lead to deformed and uneven fruits (Torrey 1967; Patterson 1990). The correlation between seed number and fruit size suggests that the fertilization of more ovules stimulates fruit growth, and the amount of pollen reaching the stigma may also be a determining factor (Serrano Roldán and Guerra-Sanz 2006).

### Pollen germination, pollen tube growth, and selection

A strong correlation exists between pollen germination rate and pollen tube elongation (Salem et al. 2007; Gajanayake et al. 2011). During the progamic phase, intense pollen competition occurs within the stigma, which can serve as the basis for gametophytic selection (Mulcahy 1979). Under stressful conditions, pollen originating from genotypes better adapted to elevated temperature may be preferentially successful in fertilization (Zamir 1982; Hedhly et al. 2004, 2005; Pasonen et al. 2002).

### Temperature and carbohydrate metabolism in pollen function

High temperatures can reduce pollen quantity, alter pollen morphology, impair viability, germination capacity, and pollen tube growth rate, thereby directly limiting fruit set (Prasad et al. 2002). Koti (2005) found that high temperature reduced the number of pollen grains produced in soybean by approximately 34% compared with the control. Sato et al. (2002a, b) found that the number of released pollen grains decreased under temperatures above the optimum (28/22 °C, day/night). This caused stress in the processes of pollen development and release, resulting in less pollen reaching the site of fertilization. An increase of 2.5 °C above the optimal mean daily temperature can reduce fruit set by up to approximately 40%. Aloni et al. (2001) found that under heat stress, pollen metabolism slows down, resulting in higher sucrose and starch concentrations, while germination capacity decreases. Elevated CO_2_, however, can increase assimilate availability, thereby improving pollen germination under high-temperature conditions. The development of the male gametophyte occurs within the anthers and requires the coordinated function of multiple tissue layers, including the tapetum (Goldberg et al. 1993; Ma 2005). The developmental period around meiosis is particularly heat-sensitive; under elevated temperature, morphological abnormalities and defective pollen formation may occur (Iwahori and Takahashi 1964; Peet et al. 1998; Sato et al. 2000). In pepper, reductions in fruit set under heat stress are primarily attributed to pollen damage, and to a lesser extent to impairment of maternal tissues (Han et al. 1996; Peet et al. 1998). Carbohydrate metabolism plays a key regulatory role in pollen development (Clement et al. 1994; Pacini 1996). In the days preceding anthesis, starch reserves are typically depleted and sucrose accumulates, and heat stress can disrupt sucrose utilization and associated metabolic fluxes (Dinar and Rudich 1985; Taub et al. 2000). In certain cultivars (e.g., Mazurka), heat stress reduced pollen germination and seed number, even when fruit number remained largely unchanged, highlighting the decoupling of fruit set and seed set under stress conditions (Aloni et al. 1996).

## Hormonal regulation and an integrated model of fruit set and abortion

### Auxin-ethylene balance and the abscission zone (AZ)

In pepper, flower and fruit abortion represent major physiological constraints on fruit formation and are regulated by the abscission zone (AZ) formed in the pedicel, together with the balance between auxin and ethylene signaling (Taylor and Whitelaw 2001; Wubs et al. 2009a, b; Botton et al. 2011; Chen et al. 2022). Under high-temperature conditions (approximately 32–40 °C), flower and fruit abortion frequently increase and are associated with enhanced ethylene production and reduced indole-3-acetic acid (IAA) levels in the AZ (Huberman et al. 1997). In bell pepper, ethylene promotes, whereas auxin inhibits flower abscission; accordingly, exogenous auxin applications in some studies improved vegetative growth, fruit parameters, and pollen viability (Wien et al. 1989; Kaur et al. 2017). However, the magnitude and direction of auxin effects may vary among cultivars (Motsenbocker and Arancibia 2000).

**Fig. 1:**
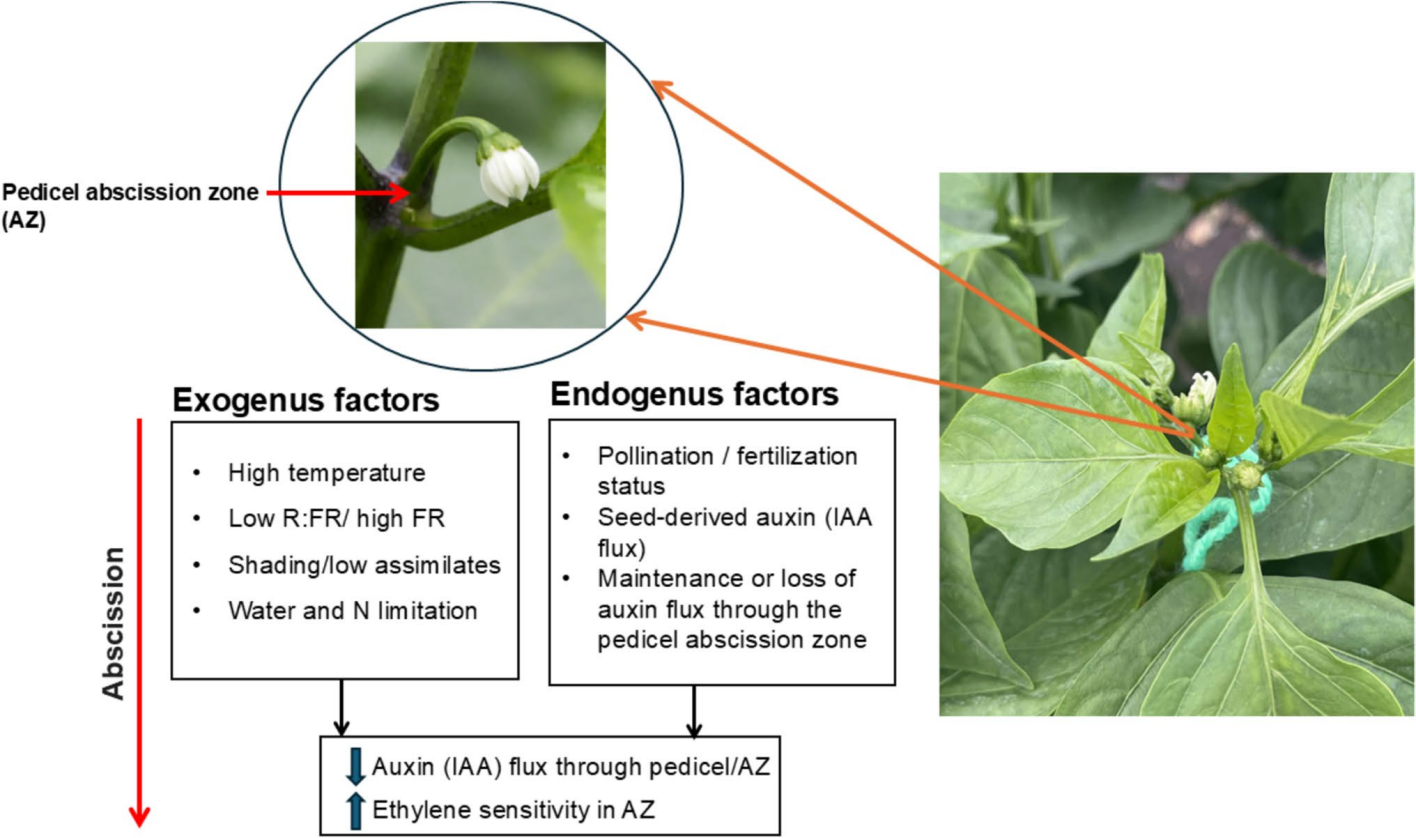
Environmental and endogenous control of flower abscission in sweet pepper: the central role of the pedicel abscission zone. Exogenous stress factors and endogenous reproductive signals converge at the pedicel abscission zone, where changes in auxin flux determine ethylene sensitivity and the decision between flower retention and abscission. Successful pollination and seed-derived auxin maintain polar auxin transport through the AZ, whereas environmental stress reduces auxin flux, enhances ethylene responsiveness, and promotes abscission

As illustrated in Fig. 1, environmental stressors and reproductive status converge at the pedicel abscission zone, where the maintenance or decline of auxin flux represents a central physiological switch between organ retention and abortion. Exogenous factors such as high temperature, altered light conditions, and resource limitation, together with endogenous signals linked to pollination and fertilization, modulate auxin transport through the pedicel and thereby determine ethylene sensitivity within the AZ, ultimately controlling whether the flower is retained or shed.

High temperatures can reduce auxin concentration and polar auxin transport in the pedicels of flowers and young fruits, while simultaneously increasing ACC levels, thereby enhancing ethylene biosynthesis (Wien et al. 1989; 1993; Huberman et al. 1997). Ethylene sensitivity of the AZ is further modulated by auxin status and carbohydrate availability, indicating close crosstalk between hormonal and metabolic signals.

Maupilé et al. (2024) showed that at 1.5 days after anthesis, fruit set in pollinated or parthenocarpic flowers is initiated by a reduction in ethylene (ET) and abscisic acid (ABA) levels, or by an increase in brassinosteroids (BR). At 3 days after anthesis, elevated auxin (IAA) and cytokinin (CK) levels stimulate gibberellin (GA) biosynthesis, thereby promoting fruit development. In contrast, in non-fertilized and non-parthenocarpic flowers, ABA and ET levels remain high after flowering, suppressing the accumulation of IAA, CK, and GA and ultimately leading to flower abscission.

### Light quality, hormone transport, and “dominance”

A low red:far-red (R:FR) ratio and increased far-red light availability can enhance flower and fruit abortion by modifying auxin biosynthesis, transport, and distribution (Küpers et al. 2020). These effects are closely linked to assimilate competition and hormonal dominance, whereby auxin export from earlier-developing fruits and the apical shoot can suppress the development and retention of later-forming flowers and fruits (Bangerth 2000; Wubs et al. 2009a, b; Walker & Bennett 2018). According to Bangerth’s (1989; 2000) “correlative-driven abscission” concept, hormonal dominance among developing sinks—primarily mediated through the auxin–ethylene balance—plays a central role in regulating abscission processes.

### Other hormones and assimilate supply

In pepper, comparatively less information is available on the role of abscisic acid (ABA); however, studies in other species indicate that ABA concentration often positively correlates with fruit senescence and may act as a mediator linking assimilate limitation to enhanced ethylene biosynthesis (Guinn & Brummett 1987; Gómez-Cadenas et al. 2000). Gibberellins are essential for fruit set, and inhibition of gibberellin biosynthesis or signaling can markedly increase the abscission of young fruits (Serrani et al. 2007; Webster & Spencer 2000). Overall, hormonal regulation and assimilate availability function as tightly interconnected and mutually reinforcing drivers of flower and fruit abortion (Guinn 1976; Wien et al., 1989b; Aloni et al. 1997).

### Ovate family proteins (OFP) and hormonal networks

Ovate Family Proteins (OFPs) are plant-specific transcriptional regulators characterized by a conserved OVATE domain and were originally identified in tomato as key regulators of fruit shape (Liu et al. 2002; Tang et al. 2018). Subsequent studies indicate that OFP proteins also play broader roles in pepper, influencing vegetative and reproductive development, floral organ morphogenesis, pollen viability, and the maintenance of male fertility (Tsaballa et al. 2011; Borovsky et al. 2021). Yin et al. (2025) demonstrated that overexpression of the pepper CaOFP20 gene in tomato resulted in style elongation, anther developmental abnormalities, and reduced male fertility, effects that were associated with altered expression of genes involved in gibberellin metabolism (Yin et al. 2025). These findings suggest that OFPs function as regulatory nodes linking developmental programs with hormonal homeostasis, thereby indirectly influencing reproductive success under stress conditions.

## Fruit drop (flower and fruit abscission): significance, mechanisms, and triggers

Low fruit set, caused by high rates of flower and young fruit abscission, is a major yield-limiting factor in sweet pepper (Wubs et al. 2009a, b; Chen et al. 2024a, b). In protected cultivation, up to 70–80% of generative organs may be shed, resulting in cyclic yield fluctuations and unstable market supply (Heuvelink et al. 2004). Abscission is an evolutionarily conserved developmental process that enables organ detachment through the integration of hormonal, environmental, and developmental cues (Estornell et al. 2013; Tonutti et al. 2023).

At the molecular level, abscission is a genetically programmed and tightly regulated process. In model systems such as *Arabidopsis thaliana*, several distinct phases can be distinguished, ranging from abscission zone (AZ) formation to cell wall remodeling and final sealing of the separation surface (Kim 2014; Kim et al. 2019). The AZ consists of specialized cell layers, in which degradation of the pectin-rich middle lamella and the formation of a protective boundary layer are key steps (Lee & Kwak 2018; Patharkar & Walker 2018).

### Triggering factors

In bell pepper, flower and fruit abscission are influenced by multiple interacting factors, including high temperature, reduced light intensity, water and salt stress, plant density, insufficient pollination, application of plant growth regulators (PGRs), and unfavorable source–sink relationships (Reyes-Castro et al. 2024). Stress conditions such as shading and high temperature can reduce photosynthetic performance and vegetative growth (Turner & Wien 1994a,b; Aloni et al. 1996). However, several studies indicate that abortion is not driven solely by reduced photosynthesis, but rather by altered dry matter partitioning and reduced assimilate supply to generative organs (Turner & Wien 1994b; Aloni et al. 1996).

Flower and fruit abortion are commonly interpreted within two conceptual frameworks: (i) competition for assimilates (source-sink limitation) and (ii) hormonal dominance mediated by auxin export and ethylene signaling (Marcelis et al. 2004; Bangerth 1989; 2000). These mechanisms are not mutually exclusive, as reduced assimilate availability can trigger hormonal responses that activate the AZ and promote abscission.

## Seedlessness and parthenocarpy: stress, hormones, and breeding value

According to Rylski & Spigelman (1982) low night temperatures (approximately 12–15 °C) can markedly reduce flowering and fruit set in pepper by impairing pollen viability, leading to flower abortion or the formation of parthenocarpic (seedless) fruits, and at a low night temperature (15 °C) most of the pepper fruits were seedless: on average, about 34% of the fruits were parthenocarpic. Polowick & Sawhney, (1985) likewise found that low day/night temperature combinations (for example, 18 °C during the day and 15 °C at night) strongly affected flower and fruit development, reduced male gamete (pollen) viability, and consequently decreased fruit set and seed number, thereby increasing the proportion of seedless fruits. Seedless fruits are often smaller and morphologically irregular, although the extent of parthenocarpy shows substantial cultivar dependence (Shifriss & Eidelman 1986; Tiwari et al. 2007).

Seedless fruit formation is frequently induced through the exogenous application of plant growth regulators, including auxins, gibberellins, and cytokinins, which can promote fruit set under stress conditions (Heuvelink & Körner 2001; Silveira et al. 1986). However, certain treatments (e.g., GA_3_) may negatively affect pollen viability and cause abnormal floral development, limiting their practical applicability (Pressman et al. 1998; Sawhney 1981).

From a biological perspective, fruit development is normally initiated by pollination and fertilization, with the primary function of the fruit being seed protection and dispersal (Lord and Russell, 2002). From an economic standpoint, however, seedless fruits may be desirable in several crops (Varoquaux et al. 2000). Hormones produced by developing seeds, particularly auxin, act as central regulators of fruit growth, making the hormonal and genetic control of parthenocarpy an important target in breeding programs (Sotelo-Silveira et al. 2014; Joldersma & Liu 2018).

## Genetics, heterosis, and male-sterility systems

### Hybrid breeding and *Capsicum* diversity

In pepper, hybrid breeding remains partially underexploited, partly due to limited understanding of heterosis and reciprocal effects. Increasing climatic uncertainty enhances the value of genetic diversity within the *Capsicum* genus for developing resilient and market-adapted cultivars (Tripodi et al. 2020; Devi et al. 2021). Of the 41 described *Capsicum* species, only five are domesticated; domestication and selection have generated extraordinary diversity in fruit morphology and quality traits (Pickersgill 1997; Barboza et al. 2019). Although many *Capsicum* species are predominantly self-pollinating, heterosis has been investigated only to a limited extent, despite reports of increased yield, biomass accumulation, and altered gene expression in selected hybrids (Garcés-Claver et al. 2007; Singh et al. 2014; Yang et al. 2021).

### Male sterility (GMS and CMS/CGMS) in seed production

Genetic male sterility (GMS) represents a key tool in *Capsicum* hybrid breeding, as it can substantially reduce labor costs and prevent self-pollination (Meena et al. 2018; Jeong et al. 2018). GMS is often controlled by single recessive nuclear genes (Swamy et al. 2017), allowing efficient introgression into breeding lines using molecular markers (Dhaliwal & Jindal 2014a, b; Zhang et al. 2020). The genetic basis of cytoplasmic-genic male sterility (CMS/CGMS) was first described by Peterson (1958); in pepper, sterility is commonly induced by the interaction of sterile cytoplasm (S) with a recessive nuclear gene (*Rf1*), while fertility restoration depends on a dominant *Rf1* allele (Shifriss 1997). In some genotypes, an additional recessive gene (*Rf2*) contributes to complete male sterility (Novak et al. 1971).

## Water and nutrient supply, humidity: abortion and seed set

### Nitrogen supply and abscission

In bell pepper, flower abscission can be higher under low nitrate (NO_3_⁻) levels; correlations have been reported between growth parameters and the plant’s carbon and nitrogen content (Riga 2014). Increasing total nitrogen (N) concentration (3 → 6 → 9 mM) enhanced flower and fruit set; in some systems, a partial ammonium proportion during the vegetative stage was beneficial, whereas nitrate dominance during fruit development proved more favorable (Xu et al. 2001).

Flower and fruit abortion increases linearly with decreasing photoassimilate availability and may be triggered by leaf pruning, high plant density, reduced radiation, or rapid growth of early-developing fruits (Marcelis et al. 2004). The most sensitive window typically occurs in the weeks surrounding anthesis.

### Relative humidity and temperature

Baër and Smeets (1978) reported that fruit drop rates were largely unaffected by constant relative humidity (RH) levels of 55%, 80%, or 95%; however, at 95% RH, fruit set was reduced. Other studies observed negative correlations between flower and fruit number and nighttime RH, while daytime RH also influenced fruit drop (Bakker 1989). At high temperatures (≈33 °C), Erickson and Markhart (2001) found no strong RH effects on flower buds and flowers, suggesting that heat-induced abortion is not solely driven by humidity conditions.

### Water supply and cultivar effects

Under water deficit, some cultivars (e.g., ‘Blue Star’) exhibit pronounced increases in flower and fruit abortion (Jaafar et al. 1994), whereas others show moderate or non-significant responses (González-Dugo et al. 2007; Fernández et al. 2005). Increasing irrigation frequency has been shown to promote earlier flowering and higher yield in several experiments (Sezen et al. 2006; Tanaskovik et al. 2015). Water stress can reduce the concentration of reducing sugars in flower buds, potentially impairing pollination and enhancing flower drop (Ningoji et al. 2024).

## Conclusions and recommendations

The ecological and evolutionary consequences of climate change are likely to manifest through the high sensitivity of reproductive processes. Sexual reproduction is central to plant fitness, and environmental stress during gametogenesis and fertilization can influence adaptation across generations via gametophytic selection and phenotypic plasticity. Epigenetic mechanisms, including DNA methylation, may contribute to the persistence of stress effects and, in some cases, to transgenerational responses. Because both pollen and the female gametophyte are directly exposed to environmental conditions, the reproductive phase represents a critical vulnerability point, where stress-induced fertility reduction acts both as a risk factor and as a potential selective filter.

Fruit set in pepper and the success of hybrid seed production emerge from the tight integration of environmental, physiological, hormonal, and genetic processes. Temperature and light conditions directly affect pollen development, viability, and fertilization success, while light intensity and spectral composition modulate the retention of generative organs through photoassimilate availability and photoreceptor-mediated signaling. At the organ level, the auxin-ethylene balance, together with associated ABA and gibberellin pathways, functions as a critical regulatory switch within the abscission zone. These hormonal signals interact closely with source-sink dynamics and correlative dominance, providing a mechanistic explanation for cyclic patterns of fruit set observed under stress conditions. At the genetic level, diversity within the *Capsicum* gene pool, together with the application of GMS and CMS/CGMS systems, underpins the efficiency, genetic purity, and economic sustainability of hybrid seed production.

Looking ahead, progress in stress-resilient pepper breeding will increasingly depend on the integration of physiological insight with applied breeding strategies. Key advances are expected from the empirical linkage of rapid, pollen-based stress phenotyping to fruit-set and seed-set performance, the targeted optimization of light spectrum and intensity in protected cultivation, and the reliable marker-assisted deployment of male-sterility systems. In parallel, improved strategies for the introgression of adaptive alleles from wild *Capsicum* relatives, while minimizing linkage drag, will be essential. Together, these approaches provide a foundation for developing robust, climate-adapted hybrid seed production systems capable of maintaining yield stability under increasing environmental variability.

## Data Availability

Not applicable.

## Abbreviations

ABA: Abscisic acid
ACC: 1-Aminocyclopropane-1-carboxylic acid
AZ: Abscission zone
B:R: Blue:red light ratio
CK: Cytokinin
CMS: Cytoplasmic male sterility
CGMS: Cytoplasmic-genic male sterility
ET: Ethylene
FR: Far-red light
GA: Gibberellin
GMS: Genetic male sterility
IAA: Indole-3-acetic acid
INV: Invertase
OFP: Ovate family protein
PGR: Plant growth regulator
PSS: Phytochrome photostationary state
QTL: Quantitative trait locus
R:FR: Red:far-red light ratio
ROS: Reactive oxygen species
sHSP: Small heat shock protein

## References

[CR1] Agnihotri MS, Singh BP (1975) Pollen production and allergenic significance of some grasses around Lucknow. J Palynol 11:151–154

[CR2] Aguilar MA, Morrell PL, Roose ML, Kim S-C (2009) Genetic diversity and structure in semiwild and domesticated chiles (*Capsicum annuum; Solanaceae*) from Mexico. Am J Bot 96:1190–1202 21628269 10.3732/ajb.0800155

[CR3] Alghabari F, Ihsan MZ, Hussain S, Aishia G, Daur I (2015) Effect of Rht alleles on wheat grain yield and quality under high temperature and drought stress during booting and anthesis. Environ Sci Pollut Res 22:15506–15515. 10.1007/s11356-015-4724-z 10.1007/s11356-015-4724-z 26006072

[CR4] Aloni B, Karni L, Zaidman Z, Schaffer AA (1996) Changes of carbohydrates in pepper (*Capsicum annuum* L.) flowers in relation to their abscission under different shading regimes. Ann Bot 78:163–168

[CR5] Aloni B, Karni L, Zaidman Z, Schaffer AA (1997) The relationship between sucrose supply, sucrose-cleaving enzymes and flower abortion in pepper. Ann Bot 79:601–605

[CR6] Aloni B, Peet M, Pharr M, Karni L (2001) The effect of high temperature and high atmospheric CO2 on carbohydrate changes in bell pepper (*Capsicum annuum*) pollen in relation to its germination. Physiol Plant 112:505–512 11473710 10.1034/j.1399-3054.2001.1120407.x

[CR8] Ascough GD, Nogemane N, Mtshali NP, Van Staden J (2005) Flower abscission: environmental control, internal regulation and physiological response of plants. S Afr J Bot 71:287–301

[CR9] Baër J, Smeets L (1978) Effect of relative humidity on fruit set and seed set in pepper (*Capsicum annuum* L.). Neth J Agric Sci 26:59–63

[CR10] Bakker JC (1989) The effects of air humidity on flowering, fruit set, seed set, and fruit growth of glasshouse sweet pepper (*Capsicum annuum* L.). Sci Hortic 40:1–8

[CR11] Bangerth F (1989) Dominance among fruits/sinks and the search for a correlative signal. Physiol Plant 76:608–614

[CR12] Bangerth F (2000) Abscission and thinning of young fruit and their regulation by plant hormones and bioregulators. Plant Growth Regul 31:43–59

[CR13] Barboza GE, García CC, González SL, Scaldaferro M, Reyes X (2019) Four new species of Capsicum (*Solanaceae*) from the tropical Andes and an update on the phylogeny of the genus. PLoS ONE 14:e0209792 30650102 10.1371/journal.pone.0209792 PMC6334993

[CR14] Bergonia EA, Cervancia CR (1992) Comparison of honey bee and hand pollination of cucumber (*Cucumis sativus* L.). Philipp J Sci 121:255–262

[CR15] Borovsky Y, Raz A, Doron-Faigenboim A, Zemach H, Karavani E, Paran I (2021) Pepper fruit elongation is controlled by *Capsicum annuum* OVATE family protein 20. Front Plant Sci 12:815589 35058962 10.3389/fpls.2021.815589 PMC8763684

[CR16] Botton A, Eccher G, Forcato C, Ferrarini A, Begheldo M, Zermiani M, Moscatello S, Battistelli A, Velasco R, Ruperti B, Ramina A (2011) Signaling pathways mediating the induction of apple fruitlet abscission. Plant Physiol 155:185–208 21037112 10.1104/pp.110.165779 PMC3075760

[CR17] Carrizo García C, Barfuss MHJ, Sehr EM, Barboza GE, Samuel R, Moscone EA, Ehrendorfer F (2016) Phylogenetic relationships, diversification and expansion of chili peppers (*Capsicum, Solanaceae*). Ann Bot 118:35–51 27245634 10.1093/aob/mcw079 PMC4934398

[CR18] Chen S, Marcelis LFM, Heuvelink E (2022) Far-red radiation increases flower and fruit abortion in sweet pepper (*Capsicum annuum* L.). Sci Hortic 305:111386. 10.1016/j.scienta.2022.111386

[CR19] Chen S, Marcelis LFM, Offringa R, Kohlen W, Heuvelink E (2024a) Far-red light-enhanced apical dominance stimulates flower and fruit abortion in sweet pepper. Plant Physiol 195:kiae088. 10.1093/plphys/kiae088 10.1093/plphys/kiae088 PMC11142340 38366641

[CR20] Chen S, Dalla Villa V, Kohlen W, Kusuma P, Offringa R, Marcelis LFM, Heuvelink E (2024b) High ratio of blue:red light reduces fruit set in sweet pepper, which is associated with low starch content and hormonal changes. Environ Exp Bot 225:105850

[CR21] Cheng J, Qin C, Tang X, Zhou H, Hu Y, Zhao Z, Cui J, Li B, Wu Z, Yu J et al (2016) Development of a SNP array and its application to genetic mapping and diversity assessment in pepper (*Capsicum* spp.). Sci Rep 6:33293 27623541 10.1038/srep33293 PMC5020730

[CR22] Cheng Q, Wang P, Liu J, Wu L, Zhang Z, Li T, Gao W, Yang W, Sun L, Shen H (2018) Identification of candidate genes underlying genic male-sterile msc-1 locus via genome resequencing in *Capsicum annuum* L. Theor Appl Genet 131:1861–1872. 10.1007/s00122-018-3119-1 29855672 10.1007/s00122-018-3119-1

[CR23] Clement C, Chavant L, Burrus M, Audran JC (1994) Anther starch variation in lilium during pollen development. Sex Plant Reprod 7:347–356

[CR24] Crane JC (1969) The role of hormones in fruit set and development. HortScience 4:108–111

[CR25] Dafni A (1992) Pollination ecology: a practical approach. Oxford University Press, New York

[CR26] Demers D-A, Gosselin A, Wien HC (1998) Effects of supplemental light duration on greenhouse sweet pepper plants and fruit yields. J Am Soc Hortic Sci 123:202–207

[CR27] Deprá MS, Delaqua GG, Freitas L, Gaglianone MC (2014) Pollination deficit in open-field tomato crops (*Solanum lycopersicum* L., *Solanaceae*) in Rio de Janeiro state, Southeast Brazil. J Pollinat Ecol 12:1–8. 10.26786/1920-7603(2014)7

[CR28] Devi J, Sagar V, Kaswan V, Ranjan JK, Kumar R, Mishra GP, Dubey RK, Verma RK (2021) Advances in breeding strategies of bell peppers (*Capsicum annuum*). In: Al-Khayri JM, Jain SM, Johnson DV (eds) Advances in Plant Breeding Strategies: Vegetable Crops, vol 9. Springer International Publishing, Cham, pp 3–58

[CR29] Dhaliwal MS, Jindal SK (2014a) Genetic male sterility in chilli pepper. Plant Breed 133:29–37

[CR30] Dhaliwal MS, Jindal SK (2014b) Induction and exploitation of nuclear and cytoplasmic male sterility in pepper (Capsicum spp.): a review. J Hortic Sci Biotechnol 89:471–479. 10.1080/14620316.2014.11513108

[CR31] Dhall RD, Hundal JS, Saxena A (2011) Floral biology studies in chili (*Capsicum annuum* L.). Veg Sci 38:221–224

[CR32] Dickson MH (1992) Breeding for heat tolerance in green beans and broccoli. In: Kuo CG (ed) Adaptation of food crops to temperature and water stress. AVRDC, Shanhua, Taiwan, pp 296–302

[CR33] Dinar M, Rudich J (1985) Effect of heat stress on assimilate partitioning in tomato. Ann Bot 56:239–248

[CR34] Driedonks N et al (2018a) Natural variation for heat tolerance. Plant Physiol 176:118–135

[CR35] Driedonks N, Wolters-Arts M, Huber H, de Boer GJ, Vriezen W, Mariani C, Rieu I (2018b) Exploring the natural variation for reproductive thermotolerance in wild tomato species. Euphytica 214:67

[CR36] Erickson AN, Markhart AH (2001) Flower production, fruit set, and physiology of bell pepper during elevated temperature and vapor pressure deficit. J Am Soc Hortic Sci 126:697–702

[CR37] Estornell LH, Agustí J, Merelo P, Talón M, Tadeo FR (2013) Elucidating mechanisms underlying organ abscission. Plant Sci 199:48–60. 10.1016/j.plantsci.2012.10.008 23265318 10.1016/j.plantsci.2012.10.008

[CR38] FAO, 2025. FAOSTAT (Quality / QCL data). Available at: https://www.fao.org/faostat/en/#data/QCL (Accessed 28 Aug 2025)

[CR39] Fernández MD, Gallardo M, Bonachela S, Orgaz F, Thompson RB, Fereres E (2005) Water use and production of a greenhouse pepper crop under optimum and limited water supply. J Hortic Sci Biotechnol 80:87–96

[CR40] Ferree DC, McArtney SJ, Scurlock DM (2001) Influence of irradiance and period of exposure on fruit set of French-American hybrid grapes. J Am Soc Hortic Sci 126:283–290

[CR41] Firon N, Shaked R, Peet MM, Pharr DM, Zamski E, Rosenfeld K, Althan L, Pressman E (2006) Pollen grains of heat tolerant tomato cultivars retain higher carbohydrate concentration under heat stress conditions. Sci Hortic 109:212–217

[CR42] Fu D, Xiao M, Hayward A, Fu Y, Liu G, Jiang G, Zhang H (2014) Utilization of crop heterosis: a review. Euphytica 197:161–173. 10.1007/s10681-014-1103-7

[CR43] Gajanayake B, Trader BW, Raja Reddy K, Harkess RL (2011) Screening ornamental pepper cultivars for temperature tolerance using pollen and physiological parameters. HortScience 46:878–884

[CR44] Garcés-Claver A, Gil-Ortega R, Alvarez-Fernández A, Arnedo-Andrés MS (2007) Inheritance of capsaicin and dihydrocapsaicin, determined by HPLC-ESI/MS, in an intraspecific cross of *Capsicum annuum* L. J Agric Food Chem 55:6951–6957 17661486 10.1021/jf070951x

[CR45] Giorno F, Wolters-Arts M, Mariani C, Rieu I (2013) Ensuring reproduction at high temperatures: the heat stress response during anther and pollen development. Plants Basel 2:489–506 27137389 10.3390/plants2030489 PMC4844380

[CR46] Gisbert-Mullor R, Padilla YG, Martínez-Cuenca MR, Lopez-Galarza S, Calatayud A (2021) Suitable rootstocks can alleviate the effects of heat stress on pepper plants. Sci Hortic 290:110529. 10.1016/j.scienta.2021.110529

[CR47] Goldberg RB, Beals TP, Sanders PM (1993) Anther development: basic principles and practical applications. Plant Cell 5(10):1217–1229 8281038 10.1105/tpc.5.10.1217 PMC160355

[CR48] Gómez-Cadenas A, Mehouachi J, Tadeo FR, Primo-Millo E, Talon M (2000) Hormonal regulation of fruitlet abscission induced by carbohydrate shortage in citrus. Planta 210:636–643 10787058 10.1007/s004250050054

[CR49] González-Dugo V, Orgaz F, Fereres E (2007) Responses of pepper to deficit irrigation for paprika production. Sci Hortic 114:77–82

[CR50] Grover A, Mittal D, Negi M, Lavania D (2013) Generating high temperature tolerant transgenic plants: achievements and challenges. Plant Sci 205–206:38–47 23498861 10.1016/j.plantsci.2013.01.005

[CR51] Guinn G (1976) Nutritional and ethylene evolution by young cotton bolls. Crop Sci 16:89–91

[CR52] Guinn G, Brummett DL (1987) Concentrations of abscisic acid and indoleacetic acid in cotton fruits and their abscission zones in relation to fruit retention. Plant Physiol 83:199–202 16665202 10.1104/pp.83.1.199 PMC1056324

[CR53] Guo M, Yin YX, Ji JJ, Ma BP, Lu MH, Gong ZH (2014) Cloning and expression analysis of heat-shock transcription factor gene CaHsfA1 from pepper (*Capsicum annuum* L.). Genet Mol Res 13:1865–1875 24668674 10.4238/2014.March.17.14

[CR54] Han XB, Li RQ, Wang JB, Miao C (1996) Effect of heat stress on pollen development and pollen viability of pepper. Acta Hortic Sin 23:359–364

[CR55] He W, Xiao Q, Pu G, Huang X, Li Y, Shi L (2017) Effect of walnut pollen on ‘Shuangzao’ fruit quality and early fruit of several. J Hunan Agric Univ 43:266–269. 10.13331/j.cnki.jhau.2017.03.008

[CR56] Hedhly A, Hormaza JI, Herrero M (2004) Effect of temperature on pollen tube kinetics and dynamics in sweet cherry, *Prunus avium* (Rosaceae). Am J Bot 91:558–564 21653411 10.3732/ajb.91.4.558

[CR57] Hedhly A, Hormaza JI, Herrero M (2005) Variation in pollen performance as influenced by temperature and the pistil. J Evol Biol 18:1494–1502 16313462 10.1111/j.1420-9101.2005.00939.x

[CR58] Hedhly A, Hormaza JI, Herrero M (2009) Global warming and sexual plant reproduction. Trends Plant Sci 14:30–36 19062328 10.1016/j.tplants.2008.11.001

[CR59] Heidmann I, Schade-Kampmann G, Lambalk J, Ottiger M, Di Berardino M (2016) Impedance flow cytometry: a novel technique in pollen analysis. PLoS ONE 11:e0165531 27832091 10.1371/journal.pone.0165531 PMC5104384

[CR60] Herath HMSN, Rafii MY, Ismail SI, Ramlee SI (2020) Improvement of important economic traits in chilli through heterosis breeding: a review. J Hortic Sci Biotechnol 96:14–23. 10.1080/14620316.2020.1780162

[CR61] Heuvelink E, Körner O (2001) Parthenocarpic fruit growth reduces yield fluctuation and blossom-end rot in sweet pepper. Ann Bot 88:69–74

[CR62] Heuvelink E, Marcelis LFM, Körner O (2004) How to reduce yield fluctuations in sweet pepper? Acta Hortic 633:349–355

[CR63] Hosseinifard M, Stefaniak S, Ghorbani Javid M, Soltani E, Wojtyla Ł, Garnczarska M (2022) Contribution of exogenous proline to abiotic stresses tolerance in plants: a review. Int J Mol Sci 23:5186. 10.3390/ijms23095186 35563577 10.3390/ijms23095186 PMC9101538

[CR64] Howarth CJ (2005) Genetic improvements of tolerance to high temperature. Abiotic stresses: plant resistance through breeding and molecular approaches. Howarth Press Inc., New York, pp 277–300

[CR65] Huberman M, Riov J, Aloni B, Goren R (1997) Role of ethylene biosynthesis and auxin content and transport in high temperature-induced abscission of pepper reproductive organs. J Plant Growth Regul 16:129–135

[CR66] Iwahori S, Takahashi K (1964) High temperature injuries in tomato. III. Effect of high temperature on flower buds of different stages of development. J Jpn Soc Hortic Sci 33:67–74

[CR67] Jaafar H, Black CR, Atherton JG (1994) Water relations, dry matter distribution and reproductive development of sweet pepper (*Capsicum annuum*). Aspects Appl Biol 38:229–306

[CR68] Jagadish SVK et al (2010a) Reproductive failure of rice under heat stress. J Exp Bot 61:4137–4148

[CR69] Jagadish SV, Muthurajan R, Oane R, Wheeler TR, Heuer S, Bennett J, Craufurd PQ (2010b) Physiological and proteomic approaches to address heat tolerance during anthesis in rice (*Oryza sativa* L.). J Exp Bot 61:143–156 19858118 10.1093/jxb/erp289 PMC2791117

[CR70] Jagadish SVK et al (2016) Genetic strategies for heat tolerance. Plant Physiol 172:731–745

[CR71] Jeong K, Choi D, Lee J (2018) Fine mapping of the genic male-sterile ms1 gene in *Capsicum annuum* L. Theor Appl Genet 131:183–191. 10.1007/s00122-017-2995-0 29032401 10.1007/s00122-017-2995-0

[CR72] Jia H, Liang X, Zhang L, Zhang J, Sapey E, Liu X et al (2022) Improving ultra-low temperature preservation technologies of soybean pollen for off-season and off-site hybridization. Front Plant Sci 13:920522. 10.3389/fpls.2022.920522 35845709 10.3389/fpls.2022.920522 PMC9280911

[CR73] Joldersma D, Liu Z (2018) The making of virgin fruit: genetic basis of parthenocarpy. J Exp Bot 69:955–962 29325151 10.1093/jxb/erx446 PMC6018997

[CR74] Kafizadeh N, Carapetian J, Kalantari KM (2008) Effects of heat stress on pollen viability and pollen tube growth in pepper. Res J Biol Sci 10:1159–1162

[CR75] Kaur S, Ghai N, Jindal SK (2017) Improvement of growth characteristics and fruit set in bell pepper (*Capsicum annuum* L.) through IAA application. Indian J Plant Physiol 22:213–220. 10.1007/s40502-017-0293-0

[CR76] Khoury CK, Carver D, Barchenger DW, Barboza G, van Zonneveld M, Jarret R, Bohs L, Kantar M, Uchanski M, Mercer K et al (2020) Modeled distributions and conservation status of the wild relatives of chile peppers (*Capsicum* L.). Divers Distrib 26:209–225

[CR77] Kim J (2014) Four shades of detachment: regulation of floral organ abscission. Plant Signal Behav 9:e976154 25482787 10.4161/15592324.2014.976154 PMC4623469

[CR78] Kim DH, Kim BD (2005) Development of SCAR markers for early identification of cytoplasmic male sterility genotype in chili pepper. Mol Cells 20:416–422 16404158

[CR79] Kim DH, Kim BD (2006) The organization of mitochondrial atp6 gene region in male-fertile and CMS lines of pepper. Curr Genet 49:59–67 16328502 10.1007/s00294-005-0032-3

[CR80] Kim J, Chun JP, Tucker ML (2019) Transcriptional regulation of abscission zones. Plants 8:154. 10.3390/plants8060154 31174352 10.3390/plants8060154 PMC6631628

[CR81] Kong Y, Stasiak M, Dixon MA, Zheng Y (2018) Blue light associated with low phytochrome activity can promote elongation growth as shade-avoidance response: a comparison with red light in four bedding plant species. Environ Exp Bot 155:345–359

[CR82] Kong Y, Schiestel K, Zheng Y (2019) Maximum elongation growth promoted as a shade-avoidance response by blue light is related to deactivated phytochrome: a comparison with red light in four microgreen species. Can J Plant Sci 100:314–326

[CR83] Kotak S, Larkindale J, Lee U, von Koskull-Döring P, Vierling E, Scharf KD (2007) Complexity of the heat stress response in plants. Curr Opin Plant Biol 10:310–316 17482504 10.1016/j.pbi.2007.04.011

[CR84] Koti S (2005) Interactive effects of carbon dioxide, temperature, and ultraviolet-B radiation on soybean (*Glycine max* L.) flower and pollen morphology, pollen production, germination, and tube lengths. J Exp Bot 56:725–736 15611147 10.1093/jxb/eri044

[CR85] Krell A (2018) Successful pollination with enhanced pollinator abundance. In: Roubik DW (ed) *The pollination of cultivated plants: a compendium for practitioner*, Vol. 2. FAO, Panama, pp 1–9

[CR86] Küpers JJ, Oskam L, Pierik R (2020) Photoreceptors regulate plant developmental plasticity through auxin. Plants 9:940 32722230 10.3390/plants9080940 PMC7463442

[CR87] Larsen DH, Li H, Shrestha S, Verdonk JC, Nicole C, Marcelis LF, Woltering EJ (2022) Lack of blue light regulation of antioxidants and chilling tolerance in basil. Front Plant Sci 13:852654 35463427 10.3389/fpls.2022.852654 PMC9021895

[CR88] Lee Y, Kwak JM (2018) Cellular coordination controlling organ separation and surface integrity in plants. BMB Rep 51:317–318. 10.5483/bmbrep.2018.51.7.142 29966583 10.5483/BMBRep.2018.51.7.142 PMC6089864

[CR89] Legris M, Ince YÇ, Fankhauser C (2019) Molecular mechanisms underlying phytochrome-controlled morphogenesis in plants. Nat Commun 10:1–15 31745087 10.1038/s41467-019-13045-0 PMC6864062

[CR90] Li Q, Chai L, Tong N, Yu H, Jiang W (2022) Potential carbohydrate regulation mechanism underlying starvation-induced abscission of tomato flower. Int J Mol Sci 23:1952. 10.3390/ijms23041952 35216070 10.3390/ijms23041952 PMC8876634

[CR91] Lin TH, Lin SW, Wang YW, van Zonneveld M, Barchenger DW (2021) Growing environment and heat treatment effects on intra- and interspecific pollination in chile pepper (*Capsicum* spp.). Agronomy 11:1275. 10.3390/agronomy11071275

[CR92] Liu J, Van Eck J, Cong B, Tanksley SD (2002) A new class of regulatory genes underlying the cause of pear-shaped tomato fruit. Proc Natl Acad Sci USA 99:13302–13306 12242331 10.1073/pnas.162485999 PMC130628

[CR188] Lord EM, Russell SD (2002) The mechanisms of pollination and fertilization in plants. Annu Rev Cell Dev Biol 18:81–105. 10.1146/annurev.cellbio.18.012502.083438 12142268 10.1146/annurev.cellbio.18.012502.083438

[CR93] Lucas MR, Ehlers JD, Huynh BL, Diop NN, Roberts PA, Close TJ (2013) Markers for breeding heat-tolerant cowpea. Mol Breed 31:529–536

[CR94] Ma H (2005) Molecular genetic analyses of microsporogenesis and microgametogenesis in flowering plants. Annu Rev Plant Biol 56:393–434 15862102 10.1146/annurev.arplant.55.031903.141717

[CR95] Marcelis LFM, Heuvelink E, Baan Hofman-Eijer LR, Den Bakker J, Xue LB (2004) Flower and fruit abortion in sweet pepper in relation to source and sink strength. J Exp Bot 55:2261–2268 15333643 10.1093/jxb/erh245

[CR96] Maupilé L, Chaib J, Boualem A, Bendahmane A (2024) Parthenocarpy, a pollination-independent fruit set mechanism to ensure yield stability. Trends Plant Sci 29(11):1254–1265. 10.1016/j.tplants.2024.06.007 39034223 10.1016/j.tplants.2024.06.007

[CR97] Meena OP, Dhaliwal MS, Jindal SK (2018) Development of cytoplasmic male sterile lines in chilli (*Capsicum annuum* L.) and their evaluation across multiple environments. Breed Sci 68:404–412. 10.1270/jsbbs.17150 30369814 10.1270/jsbbs.17150 PMC6198902

[CR98] Meena OP, Dhaliwal MS, Jindal SK (2020) Heterosis breeding in chilli pepper by using cytoplasmic male sterile lines for high-yield production with special reference to seed and bioactive compound content under temperature stress regimes. Sci Hortic 262:109036. 10.1016/j.scienta.2019.109036

[CR99] Min W (2009) *Molecular Genetic Analysis and Allelic Discrimination of the Restorer-of-fertility (Rf) Gene in Peppers* (Dissertation). Seoul National University, Seoul

[CR100] Minnaar C, Anderson B, De Jager ML, Karron JD (2019) Plant-pollinator interactions along the pathway to paternity. Ann Bot 123:225–245 30535041 10.1093/aob/mcy167 PMC6344347

[CR101] Motsenbocker CEA, RA, (2000) Tabasco pepper flower abscission at the pedicel stem zone. Acta Hortic. 10.17660/ActaHortic.2000.514.24

[CR102] Mulcahy DL (1979) The rise of angiosperms: a genecological factor. Science 206:20–23 17812428 10.1126/science.206.4414.20

[CR103] Mumtaz MA, Zhou Y, Gao C, Kamran HM, Altaf MA, Hao Y, Wang Z (2023) Interaction between transcriptional activator BRI1-EMS-SUPPRESSOR 1 and HSPs regulates heat stress tolerance in pepper. Environ Exp Bot 211:105341. 10.1016/j.envexpbot.2023.105341

[CR104] Müller F, Rieu I (2016) Acclimation to high temperature during pollen development. Trends Plant Sci 21:65–74 10.1007/s00497-016-0282-x PMC4909792 27067439

[CR105] Naves ER, Scossa F, Araújo WL, Nunes-Nesi A, Fernie AR, Zsögön A (2022) Heterosis and reciprocal effects for agronomic and fruit traits in *Capsicum* pepper hybrids. Sci Hortic 295:110821. 10.1016/j.scienta.2021.110821

[CR106] Nielsen TH, Skjærbæk HC, Karlsen P (1991) Carbohydrate metabolism during fruit development in sweet pepper (*Capsicum annuum*) plants. Physiol Plant 82:311–319

[CR107] Ningoji SN, Thimmegowda MN, Mudalagiriyappa Vasanthi BG, Shivaramu HS, Hegde M (2024) Effect of automated sensor-driven irrigation and fertigation on green pepper (*Capsicum annuum L.*) growth, phenology, quality and production. Sci Hortic. 10.1016/j.scienta.2024.113306

[CR108] Novak FJ, Betlach J, Dubovsky J (1971) Cytoplasmic male sterility in sweet pepper (*Capsicum annuum* L.) I. phenotype and inheritance of male sterile character. Z Pflanzenzucht 65:129–140

[CR109] Opeña RT, Chen JT, Kuo CG, Chen HM (1992) Genetic and physiological aspects of tropical adaptation in tomato. In: Kuo CG (ed) Adaptation of food crops to temperature and water stress. AVRDC, Shanhua, Taiwan, pp 257–270

[CR110] Oyama K, Hernández-Verdugo S, Sánchez C, González-Rodríguez A, Sánchez-Peña P, Garzón-Tiznado JA, Casas A (2006) Genetic structure of wild and domesticated populations of *Capsicum annuum* (*Solanaceae*) from northwestern Mexico analyzed by RAPDs. Genet Resour Crop Evol 53:553–562

[CR111] Pacini E (1996) Types and meaning of pollen carbohydrate reserves. Sex Plant Reprod 9:362–366

[CR112] Parry C, Wang YW, Lin SW, Barchenger DW (2021) Reproductive compatibility in *Capsicum* is not necessarily reflected in genetic or phenotypic similarity between species complexes. PLoS ONE 16:e0243689 33760824 10.1371/journal.pone.0243689 PMC8508556

[CR113] Pasonen H-L et al (2002) Genotype–environment interactions in pollen competitive ability in an anemophilous tree, *Betula pendula* Roth. Theor Appl Genet 105:465–473 12582552 10.1007/s00122-002-0944-y

[CR114] Patharkar OR, Walker JC (2018) Advances in abscission signaling. J Exp Bot 69:733–740. 10.1093/jxb/erx256 28992277 10.1093/jxb/erx256

[CR115] Patterson KJ (1990) Effects of pollination on fruit set, size, and quality in feijoa (*Acca sellowiana* (Berg) Burret). N Z J Crop Hortic Sci 18:127–131

[CR116] Peet MM, Sato S, Gardner RG (1998) Comparing heat stress effects on male-fertile and male-sterile tomatoes. J Am Soc Hortic Sci 123:191–197

[CR117] Peña-Yam LP, Muñoz-Ramírez LS, Avilesviñas SA, Flick AC, Antonio AG, Buzzy NS (2019) Floral biology studies in habanero pepper (*Capsicum chinense Jacq*.) to implement in a crossbreeding program. Agriculture. 10.3390/agriculture9120249

[CR118] Pérez-Jiménez M, Piñero MC, del Amor FM (2019) Heat shock, high CO_2_ and nitrogen fertilization effects in pepper plants submitted to elevated temperatures. Sci Hortic 244:322–329. 10.1016/j.scienta.2018.09.072

[CR119] Peterson PA (1958) Cytoplasmically inherited male sterile in *Capsicum*. Am Nat 92:111–119

[CR120] Pickersgill B (1997) Genetic resources and breeding of *Capsicum* spp. Euphytica 96:129–133

[CR121] Polowick PL, Sawhney VK (1985) Temperature effects on male fertility and flower and fruit development in *Capsicum annuum* L. Sci Hortic 25:117–127

[CR122] Prasad PVV et al (2002) Effects of elevated temperature and carbon dioxide on seed-set and yield of kidney bean (*Phaseolus vulgaris* L.). Glob Change Biol 8:710–721

[CR123] Pressman E, Moshkovitch H, Rosenfeld K, Shaked R, Gamliel B, Aloni B (1998) Influence of low night temperatures on sweet pepper flower quality and the effect of repeated pollinations, with viable pollen, on fruit setting. J Hortic Sci Biotechnol 73:131–136

[CR124] Pressman E, Peet MM, Pharr DM (2002a) The effect of heat stress on tomato pollen characteristics is associated with changes in carbohydrates concentration in developing anthers. Ann Bot 90:631–636 12466104 10.1093/aob/mcf240 PMC4240456

[CR125] Pressman E et al (2002b) The effect of heat stress on tomato pollen characteristics. J Am Soc Hortic Sci 127:265–270

[CR127] Ren R, Li Z, Li B, Xu J, Jiang X, Liu Y et al (2019) Changes of pollen viability of ornamental plants after long-term preservation in a cryopreservation pollen bank. Cryobiology 89:14–20. 10.1016/j.cryobiol.2019.07.001 31276669 10.1016/j.cryobiol.2019.07.001

[CR128] Reyes-Castro R, Núñez-Palenius HG, Valiente-Banuet JI, Sosa-Morales ME, Orosco-Alcalá BE, Guzmán-Mendoza R, Ruiz-Aguilar GML, Costilla Salazar R (2024) Organ abscission in plants: with special emphasis on bell pepper. Phyton 10:051644. 10.32604/phyton.2024.051644

[CR129] Riga P (2014) Flower abscission in pepper plants grown under different regimes of nitrogen fertilization and photosynthetically active radiation. J Plant Nutr 37:907–927. 10.1080/01904167.2013.873464

[CR130] Ruan Y-L, Patrick JW, Bouzayen M, Osorio S, Fernie AR (2012) Molecular regulation of seed and fruit set. Trends Plant Sci 17:656–665 22776090 10.1016/j.tplants.2012.06.005

[CR131] Rylski I, Spigelman M (1982) Effects of different diurnal temperature combination on fruit set of sweet pepper. Sci Hortic 17:101–106

[CR132] Salem MA, Kakani VG, Koti S, Reddy KR (2007) Pollen-based screening of soybean genotypes for high temperatures. Crop Sci 47:219–231

[CR133] Sato S et al (2000) Physiological factors limit fruit set of tomato (*Lycopersicon esculentum* Mill.) under chronic, mild heat stress. Plant Cell Environ 23:719–726

[CR134] Sato S et al (2002a) Determining critical pre- and post-anthesis periods and physiological processes in *Lycopersicon esculentum* Mill. exposed to moderately elevated temperatures. J Exp Bot 53:1187–1195 11971929 10.1093/jexbot/53.371.1187

[CR135] Sato S et al (2002b) Male reproductive processes under heat stress. Plant Cell Environ 25:193–202

[CR136] Sawhney VK (1981) Abnormalities in pepper (*Capsicum annuum*) flowers induced by gibberellic acid. Can J Bot 59:8–16

[CR137] Sawicki M, Aït Barka E, Clément C, Vaillant-Gaveau N, Jacquard C (2015) Cross-talk between environmental stresses and plant metabolism during reproductive organ abscission. J Exp Bot 66:1707–1719. 10.1093/jxb/eru533 25711702 10.1093/jxb/eru533 PMC4669552

[CR138] Senthil-Kumar M, Kumar G, Srikanthbabu V, Udayakumar M (2007) Assessment of variability in acquired thermotolerance: potential option to study genotypic response and the relevance of stress genes. J Plant Physiol 164:111–125 17207553 10.1016/j.jplph.2006.09.009

[CR139] Serrani JC, Sanjuán R, Ruiz-Rivero O, Fos M, García-Martínez JL (2007) Gibberellin regulation of fruit set and growth in tomato. Plant Physiol 145:246–257 17660355 10.1104/pp.107.098335 PMC1976567

[CR140] Serrano Roldán A, Guerra-Sanz JM (2006) Quality fruit improvement in sweet pepper culture by bumblebee pollination. Sci Hortic 110:160–166

[CR141] Sezen SM, Yazar A, Eker S (2006) Effect of drip irrigation regimes on yield and quality of field grown bell pepper. Agric Water Manag 81:115–131. 10.1016/j.agwat.2005.04.002

[CR142] Shifriss C (1997) Male sterility in pepper (*Capsicum annuum* L.). Euphytica 93:83–85

[CR143] Shifriss C, Eidelman E (1986) An approach to parthenocarpy in peppers. HortScience 21:1458–1459

[CR144] Silveira HL, Aguiar L, Leitao A, Taborda ML (1986) Effects of growth regulators for fruit setting on pepper (*Capsicum annuum* L.) production. Acta Hortic 191:189–198

[CR145] Singh P, Cheema DS, Dhaliwal MS, Garg N (2014) Heterosis and combining ability for earliness, plant growth, yield and fruit attributes in hot pepper (*Capsicum annuum* L.) involving genetic and cytoplasmic-genetic male sterile lines. Sci Hortic 168:175–188

[CR146] Sita K et al (2017a) Heat stress impacts on plant reproduction. Plant Physiol Biochem 111:1–1527875742

[CR147] Sita K, Sehgal A, HanumanthaRao B, Nair RM, Vara Prasad PV, Kumar S, Gaur PM, Farooq M, Siddique KHM, Varshney RK, Nayyar H (2017b) Food legumes and rising temperatures: effects, adaptive functional mechanisms specific to reproductive growth stage and strategies to improve heat tolerance. Front Plant Sci 8:1658. 10.3389/fpls.2017.01658 29123532 10.3389/fpls.2017.01658 PMC5662899

[CR148] Sood T, Sood S, Sood VK, Anuradha AB, Kapoor S, Sood V, Kumar N (2023) Characterisation of bell pepper (*Capsicum annuum* L. var. *grossum* Sendt.) accessions for genetic diversity and population structure based on agro-morphological and microsatellite markers. Sci Hortic. 10.1016/j.scienta.2023.112308

[CR149] Sotelo-Silveira M, Marsch-Martínez N, de Folter S (2014) Unraveling the signal scenario of fruit set. Planta 239:1147–1158 24659051 10.1007/s00425-014-2057-7

[CR150] Swamy BN, Hedau NK, Chaudhari GV, Lakshmi K, Pattanayak A (2017) CMS system and its stimulation in hybrid seed production of *Capsicum annuum* L. Sci Hortic 222:175–179. 10.1016/j.scienta.2017.05.023

[CR151] Tanaskovik V, Cukaliev O, Moteva M, Jankulovska M, Markoski M, Spalevic V, Rusevski R, Bogevska Z, Davitkov M (2015) The influence of irrigation and fertilization regime on some phenological stages and earliness of pruned pepper. J Agric for 61(2):7. 10.17707/AgricultForest.61.2.01

[CR152] Tang Y, Zhang W, Yin YL, Feng P, Li HL, Chang Y (2018) Expression of OVATE family protein 8 affects epicuticular waxes accumulation in *Arabidopsis thaliana*. Bot Stud 59:12 29691677 10.1186/s40529-018-0228-8 PMC5915979

[CR153] Taub DR, Seeman JR, Coleman JS (2000) Growth in elevated CO_2_ protects photosynthesis against high-temperature damages. Plant Cell Environ 23:649–656

[CR154] Taylor JE, Whitelaw CA (2001) Signals in abscission. New Phytol 151:323–340

[CR155] Tiwari A, Dassen H, Heuvelink E (2007) Selection of sweet pepper (*Capsicum annuum* L.) genotypes for parthenocarpic fruit growth. Acta Hortic 761:135–140

[CR156] Tonutti P, Brizzolara S, Beckles DM (2023) Reducing crop losses by gene-editing control of organ developmental physiology. Curr Opin Biotechnol 81:102925. 10.1016/j.copbio.2023.102925 37003167 10.1016/j.copbio.2023.102925

[CR157] Torrey JG (1967) Development in flowering plants. The MacMillan Company, New York

[CR158] Tripodi P, Lo Scalzo R, Ficcadenti N (2020) Dissection of heterotic, genotypic and environmental factors influencing the variation of yield components and health-related compounds in chilli pepper (*Capsicum annuum*). Euphytica 216:112

[CR159] Tripodi P, Rabanus-Wallace MT, Barchi L, Kale S, Esposito S, Acquadro A, Schafleitner R, van Zonneveld M, Prohens J, Diez MJ, Börner A, Salinier J, Caromel B, Bovy A, Boyaci F, Pasev G, Brandt R, Himmelbach A, Portis E, Finkers R, Lanteri S, Paran I, Lefebvre V, Giuliano G, Stein N (2021) Global range expansion history of pepper Capsicum spp revealed by over 10,000 genebank accessions. Proc Natl Acad Sci USA 118:e2104315118. 10.1073/pnas.2104315118 34400501 10.1073/pnas.2104315118 PMC8403938

[CR160] Tsaballa A, Pasentsis K, Darzentas N, Tsaftaris AS (2011) Multiple evidence for the role of an Ovate-like gene in determining fruit shape in pepper. BMC Plant Biol 11:46 21401913 10.1186/1471-2229-11-46 PMC3069956

[CR161] Turner AD, Wien HC (1994a) Dry matter assimilation and partitioning in pepper cultivars differing in susceptibility to stress-induced bud and flower abscission. Ann Bot 73:617–622

[CR162] Turner AD, Wien HC (1994b) Photosynthesis, dark respiration and bud sugar concentrations in pepper cultivars differing in susceptibility to stress-induced bud abscission. Ann Bot 73:623–628

[CR163] Varoquaux F, Blanvillain R, Delseny M, Gallois P (2000) Less is better: new approaches for seedless fruit production. Trends Biotechnol 18:233–242 10802558 10.1016/s0167-7799(00)01448-7

[CR164] Votava EJ, Nabhan GP, Bosland PW (2002) Genetic diversity and similarity revealed via molecular analysis among and within an in situ population and ex situ accessions of chiltepín (*Capsicum annuum* var. *glabriusculum*). Conserv Genet 3:123–129

[CR165] Wahid A, Gelani S, Ashraf M, Foolad MR (2007) Heat tolerance in plants: an overview. Environ Exp Bot 61:199–223

[CR166] Walker CH, Bennett T (2018) Forbidden fruit: dominance relationships and the control of shoot architecture. Annu Plant Rev 1:217–254

[CR167] Wang XQ, Xu WH, Ma LG, Fu ZM, Deng XW, Li LJ et al (2006) Requirement of KNAT1/BP for the development of abscission zones in *Arabidopsis thaliana*. J Integr Plant Biol 48:15–26. 10.1111/j.1744-7909.2005.00085.x-i

[CR168] Wang, J., Lu, W., Tong, Y., Yang, Q., 2016. Leaf morphology, photosynthetic performance, chlorophyll fluorescence, stomatal development of lettuce (*Lactuca sativa* L.) exposed to different ratios of red light to blue light. *Front. Plant Sci.* 7, 250. 10.3389/fpls.2016.00250 PMC4785143 27014285

[CR169] Warner RM, Erwin JE (2005) Naturally occurring variation in high temperature induced floral bud abortion across *Arabidopsis thaliana* accessions. Plant Cell Environ 28:1255–1266

[CR170] Warrington IJ, Mitchell KJ (1976) The influence of blue- and red-biased light spectra on the growth and development of plants. Agric Meteorol 16:247–262

[CR171] Webster AD, Spencer JE (2000) Fruit thinning plums and apricots. Plant Growth Regul 31:101–112

[CR172] Wien HC, Turner AD, Yang SF (1989) Hormonal basis for low light intensity induced flower bud abscission of pepper. J Am Soc Hortic Sci 114:981–985

[CR173] Wien, H.C., Aloni, B., Riov, J., Goren, R., Huberman, M., Ho, C.J., 1993. Physiology of heat stress-induced abscission in pepper. In: Kuo, C.G. (Ed.), adaptation of food crops to temperature and water stress (Proc Int Symp: Taiwan). pp. 188–198.

[CR174] Wubs AM, Heuvelink E, Marcelis LFM (2009a) Abscission of reproductive organs in sweet pepper. J Exp Bot 60:3893–3904

[CR175] Wubs AM, Heuvelink E, Marcelis LFM (2009b) Abortion of reproductive organs in sweet pepper (*Capsicum annuum* L.): a review. J Hortic Sci Biotechnol 84:467–475

[CR176] Xu G, Wolf S, Kafkafi U (2001) Effect of varying nitrogen form and concentration during growing season on sweet pepper flowering and fruit yield. J Plant Nutr 24:1099–1116. 10.1081/PLN-100103806

[CR177] Yamamoto K, Sakamoto H, Momonoki YS (2011) Maize acetylcholinesterase is a positive regulator of heat tolerance in plants. J Plant Physiol 168:1987–1992. 10.1016/j.jplph.2011.06.001 21757255 10.1016/j.jplph.2011.06.001

[CR178] Yamazaki A, Hosokawa M (2019) Increased percentage of fruit set of F1 hybrid of *Capsicum chinense* during high-temperature period. Sci Hortic 243:421–427. 10.1016/j.scienta.2018.08.049

[CR179] Yamazaki A, Kitade T, Takezawa A, Nishimura K, Maki T, Nakano R, Hosokawa M (2025) Identification of a novel quantitative trait locus improving fruit set in chili pepper (*Capsicum annuum*) under high-temperature greenhouse conditions. Sci Hortic 353:114471. 10.1016/j.scienta.2025.114471

[CR180] Yang S, Zhang Z, Chen W, Li X, Zhou S, Liang C, Li X, Yang B, Zou X, Liu F et al (2021) Integration of mRNA and miRNA profiling reveals the heterosis of three hybrid combinations of *Capsicum annuum* varieties. GM Crops Food 12:224–241 33410724 10.1080/21645698.2020.1852064 PMC7808418

[CR181] Ye CR, Argayoso MA, Redona ED, Sierra SN, Laza MA, Dilla CJ, Mo Y, Thomson MJ, Chin J, Delavina CB et al (2012) Mapping QTL for heat tolerance at flowering stage in rice using SNP markers. Plant Breed 131:33–41

[CR182] Yin L, Pan L, Chen Y, Tang P, Yin Q, Wang M, Jin Y, Wang Z, Xie L, Zou X, Liu F (2025) Heterologous overexpression of CaOFP20 from pepper modulates floral development and fertility in tomato. Sci Hortic 346:114179

[CR183] Zamir D (1982) Haploid selection for low temperature tolerance of tomato pollen. Genetics 101:129–137 17246078 10.1093/genetics/101.1.129 PMC1201846

[CR184] Zhang XF, Chen B, Zhang LY, Zhang LL, Chen XH, Zhao H, Geng SS (2015) Identification of proteins associated with cytoplasmic male sterility in pepper (*Capsicum annuum* L.). S Afr J Bot 100:1–6. 10.1016/j.sajb.2015.04.010

[CR185] Zhang Z, Zhu Y, Cao Y, Yu H, Bai R, Zhao H, Zhang B, Wang L (2020) Fine mapping of the male fertility restoration gene CaRf032 in *Capsicum annuum* L. Theor Appl Genet 133:1177–1187. 10.1007/s00122-020-03540-0 31925462 10.1007/s00122-020-03540-0

[CR186] Zhang H, Zhu J, Gong Z, Zhu JK (2022) Abiotic stress responses in plants. Nat Rev Genet 23:104–119. 10.1038/s41576-021-00413-0 34561623 10.1038/s41576-021-00413-0

[CR187] Zhu H, Dardick CD, Beers EP, Callanhan AM, Xia R, Yuan R (2011) Transcriptomics of shading-induced and NAA-induced abscission in apple (*Malus domestica*) reveals a shared pathway involving reduced photosynthesis, alterations in carbohydrate transport and signaling and hormone crosstalk. BMC Plant Biol 11:138 22003957 10.1186/1471-2229-11-138 PMC3217944

